# Effect of osmotherapy with mannitol or hypertonic saline on cerebral oxygenation and metabolism in patients with intracranial hypertension after severe brain injury

**DOI:** 10.1186/cc14527

**Published:** 2015-03-16

**Authors:** T Suys, H Quintard, C Patet, M Oddo

**Affiliations:** 1 Lausanne University Hospital, Lausanne, Switzerland

## Introduction

Osmotherapy with mannitol (Man) or hypertonic saline (HTS) is currently used to treat elevated intracranial pressure after severe acute brain injury (sABI); however, their effect on cerebral oxygenation and metabolism has not been extensively evaluated.

## Methods

A retrospective analysis of a cohort of patients with sABI after traumatic brain injury (TBI) and subarachnoid hemorrhage (SAH) monitored with cerebral microdialysis (CMD), brain oxygen (PbtO_2_) and ICP, who were treated with Man (20%, 0.5 g/kg) or HTS (7.5%, 100 ml) for ICP >25 mmHg. Osmotherapy was administered over 20 minutes and each patient's individual response to intervention was analyzed up to 120 minutes following infusion. Only episodes where no other hypertonic solute was administered within 2 hours before or after treatment were selected. Variables analyzed included CMD lactate, pyruvate, glucose, glutamate, lactate/pyruvate ratio, and main brain physiologic variables ICP, PbtO_2_, CPP. Analysis was conducted using mixed-effects multilevel regression.

## Results

Sixty-four treatments (32 HTS, 32 Man) were studied among 26 patients (19 TBI, seven SAH; age 42 ± 17 years, time from injury to treatment 2.6 ± 1.9 days). Man and HTS effectively decreased ICP (coefficient = -2.5 mmHg, 95% CI = -3.2 to -1.8 mmHg and -2.9 (-3.8 to -2.0) respectively; both *P <*0.0001). No significant difference was found in CMD lactate, pyruvate, glucose and PbtO_2_ after HTS or Man treatment. CMD glutamate decreased significantly after Man (-0.73 ( -1.41 to -0.052); *P *< 0.05), but not after HTS.

## Conclusion

Osmotherapy with Man and HTS treatment had no effect on cerebral oxygenation and metabolism. Man, but not HTS, favorably reduced brain glutamate. These findings support further investigation to test the value of alternative osmotic agents (such as hypertonic lactate) that may reduce ICP and at the same time improve cerebral metabolism after sABI.

## Acknowledgements

Supported by Grants from the Swiss National Science Foundation and the Novartis Foundation for Biomedical Research.

